# Ethics of Medical Students as Embedded Simulation Participants in Ultrasound Education

**DOI:** 10.5811/westjem.68197

**Published:** 2026-10-18

**Authors:** Samantha K. Chao, Charles W. Kropf, Laura R. Hopson, Creagh Boulger, Rachel Liu, Michelle Feeney, Robert D. Huang, Nik Theyyunni, Jessica Koehler, David A. Haidar

**Affiliations:** * University of Michigan, Department of Emergency Medicine, Ann Arbor, Michigan; † The Ohio State University, Department of Emergency Medicine, Columbus, Ohio; ‡ Yale School of Medicine, Department of Emergency Medicine, New Haven, Connecticut; § Northwestern Medicine, Department of Emergency Medicine, Chicago, Illinois

## Abstract

**Introduction:**

Medical students are sometimes used as embedded simulation participants (ESP) to facilitate ultrasound training on live volunteers. There is little guidance on how to safeguard the autonomy and dignity of medical student ESPs serving in this role. Based on real student experiences, we used four cases to illustrate the ethical dilemmas that can arise when ESPs are used, including the examination of sensitive body areas, risk for coercion, discovery of incidental findings in a public and professional setting, and social media posting of training events. Through these cases, we explore and articulate the relevant ethical obligations educators have to obtain informed consent, sensitively address incidental findings, and ensure the psychological and physical safety of ESPs.

**Methods:**

We developed interventions and procedures at our institution to address the obligation to promote ESP autonomy and bodily dignity, including a comprehensive consent form, ombudsperson role, and restrictions surrounding photography and social media posting of educational events.

**Results:**

These interventions were implemented in January 2024 in the emergency department of a large academic medical center affiliated with a medical school. Since implementation, 96 ESPs have participated in 16 discrete educational events. There has been anecdotal positive reception of these interventions. Recruiting and training individuals for the ombudsperson role carried some challenges.

**Conclusion:**

Proactive measures are necessary to support the autonomy and safety of medical student ESPs. There are practical challenges associated with recruitment, training, and implementation of the ombudsperson role. We hope that other institutions who use medical student ESPs will consider incorporating similar practices to promote the psychological and bodily safety of their volunteers.

## INTRODUCTION

Ultrasonography training is increasingly being integrated into undergraduate and graduate medical education.^1^,^2^ Hands-on training is essential for learners to develop technical skills, and learners may practice on a variety of subjects, ranging from inanimate simulation models to actual patients.^3^ Some ultrasound programs invite medical students to volunteer as live human models. Experts in ultrasound education recommend using alternatives to the term “model” to maintain a professional relationship^4^; therefore, we refer to student volunteers as embedded simulation participants (ESP). In simulation literature, ESPs are defined as individuals trained or scripted to play a role in a simulated encounter to help guide the scenario.^5^,^6^ In ultrasound learning, the ESP serves as a source of learner guidance and feedback.

Existing literature describes the use of students as standardized patients in clinical skills training as a cost-effective alternative to hiring occupational or trained standardized patients.^4^,^7^–^10^ However, using students in this role can present challenges. As one study exploring the experiences of nursing students as standardized patients for clinical examination of their peers described: “They felt that examinees could infringe on their bodies at any moment and that they were continually unable to prepare for such interference.”^8^ Little guidance exists for institutions who wish to use students as ESPs to craft an effective approach that considers the students’ bodily autonomy and dignity. In ultrasound education literature, a 2014 report describing a consent process and bill of rights serves as the sole example.^4^ Furthermore, there is no literature to date that examines the underlying ethical obligations educators have to support student ESPs. Below, we describe four cases based on real student concerns at our institution. Following these cases, we describe relevant ethical principles and frameworks educators can use to obviate challenges with using medical student ESPs.

### Cases

1. A medical student ESP is assigned to the cardiac ultrasound station at a training event for emergency medicine residents. The ESP did not realize her assignment would involve scanning the chest and feels uncomfortable that this sensitive area will be the focus of the station. She is receiving financial compensation for participating that will help her meet expenses; so she is hesitant to rescind her participation completely. She is also applying into emergency medicine and is wary of the impact either participating or not participating will have on residents who could be her future colleagues. She is unsure whom to ask for help at the event.
2. A medical student ESP participates in an ultrasound training event and was not made aware of its gamified nature. They were surprised to find themself in a station with dimmed lights, loud music, and learners dressed in costume. During one of the game competitions, a distracted learner uses too much force on the probe, causing the ESP pain. At the end of the day, the ESP leaves the event feeling overwhelmed and uncomfortable with their experience.
3. A medical student ESP is being scanned at a station teaching focused assessment with sonography for trauma exams. While looking at the patient’s right upper quadrant, the facilitator of the station notices multiple cystic structures in the right kidney. The medical student was unaware that he had these findings and has a family history of polycystic kidney disease. The training event was hosted for medical students who are the ESP’s peers, and he is distressed that this incidental finding was encountered while his peers were observing.
4. After volunteering as an ESP at an ultrasound training event, a medical student finds photos posted on social media documenting the event in which their abdomen is showing despite draping. The ESP had not consented to their photograph being taken and is uncomfortable with these images being publicly posted. They are unsure who to reach out to regarding the photo.

### Informed Consent

Medical students volunteering as ESPs should provide their informed consent for participation. Similar to volunteer human research subjects, obtaining informed consent is essential to respecting an individual’s autonomy, particularly when they are participating altruistically in an intervention that may not provide direct benefit and is associated with potential risks.^11^,^12^ Obtaining informed consent is a process healthcare professionals are familiar with. The full risks and benefits of volunteering as an ESP should be explained.^13^ As in Case 1, an easily anticipated risk of acting as an ESP involves bodily exposure to facilitate ultrasound image acquisition. Another risk documented in the literature is identifying incidental findings (discussed further below). There may be some discomfort associated with the pressure from an ultrasound probe, which can be anticipated; but undue pain, as in Case 2, should be avoided.^13^ Finally, to prevent situations such as Case 4, ESPs should explicitly consent for inclusion in photographs and social media postings.

There are multiple benefits associated with participating as an ESP as well. Studies have described the passive learning and professional development ESPs experience simply by being present, observing the learners, and participating in their instruction.^4^,^7^–^10^ There is also benefit from the role-reversal in which students experience what it’s like to be a patient.^9^ Having medical students as ESPs at these events can facilitate networking and recruitment into specialties engaging with clinical applications of ultrasound. Finally, some programs offer financial compensation for an ESP’s time. This practice is discussed more below.

### Psychological Safety

Psychological safety is a concept from management sciences recently applied in medical education literature. It describes creating an environment conducive to learner risk-taking and fosters performance and growth. It is also fundamentally a construct concerned with the perceived consequences of certain interpersonal risks.^14^ Although primarily applied to circumstances such as providing learner feedback, safety reporting, and clinical performance,^14^,^15^ the concept of psychological safety and promoting it is applicable to the experience of medical student ESPs.

In Case 1, the medical student ESP is hesitant to raise concerns about the station she was assigned because she is in an environment with individuals who may be mentors, future colleagues, or future supervisors. There is no clear figure for her to turn to with her concerns. She is apprehensive of the interpersonal risk of speaking up, asking to be assigned to another station, or choosing to leave, and the potential impact any one of these actions may have on her future training and career. This is not a psychologically safe environment and provides little recourse for a medical student who is at the bottom of the power differential.

Additionally, from the case examples, setting expectations in advance would have helped prepare the ESPs for what they may experience or encounter during the event. Being informed in advance which station they were assigned to and who the learner population will be could have allowed opportunity to voice concerns. Informing the ESPs of the gamified nature of the event and potential for photography would also have been beneficial. Gamification improves outcomes by improving learning behavior and attitudes toward learning; therefore, it is a crucial part of hands-on education.^16^–^18^ However, without prebriefing, unpredictable stresses during training events, particularly those that are gamified, can create an unsafe environment for student volunteers.

### Sensitive Exams

Medical educators are charged with safeguarding the physical safety of student volunteers as much as they are charged with safeguarding their psychological safety. Invasive ultrasound exams, such as pelvic exams with an endocavitary probe, exceed what a medical student volunteer would reasonably be expected to consent to. There is the particular risk of coercion if a medical student interested in financial compensation or professional networking is offered this compensation in return for an invasive educational exam.

One ultrasound program describes the scope of allowable scanning as “G-rated modeling” and explicitly excludes examinations of the breasts, genitalia, and those that require use of an endocavitary probe.^4^ In addition to these explicitly sensitive exams, certain ultrasound examinations may require learners to scan adjacent to sensitive regions of the body. Like in Case 1, cardiac ultrasound inherently requires ultrasound examination of the chest. Similarly, duplex venous ultrasound and other studies involving the inguinal regions require examination near the groin. To best promote ESP autonomy and to avoid excessive paternalism based on gendered assumptions, ESPs should be informed of which body parts will be exposed during an exam and given the opportunity to decline a particular examination if it is not one they are comfortable with. Embedded simulation participants should also always be appropriately draped during any ultrasound examination. Educators may also encourage ESPs to drape themselves to promote bodily autonomy.

### Incidental Findings

In emergency medicine, where point-of-care ultrasonography (POCUS) is frequently used in clinical practice, resident and attending physicians have been found to encounter incidental findings on 9.4% to 26% of bedside ultrasounds.^19^,^20^ Thus, there is a reasonable likelihood an ESP may have an incidental finding while being scanned educationally. As already described in the literature, clear protocols should be proactively developed, so that facilitators know how to address incidental findings discovered in volunteer ESPs.^21^,^22^ It is important to prescan volunteers, so that these incidental findings may be encountered and discussed in a private setting; additionally, educators also have an obligation to provide guidance on next steps. This process is already codified in guidelines developed by the American Institute of Ultrasound in Medicine.^23^ In Case 3, it would be important to discuss the incidental finding, explain that it requires follow-up, and advise the ESP that he should make an appointment with his primary care physician for further evaluation. It is also important to ask the ESP whether they feel comfortable having that part of the body scanned with the incidental finding present, or if they prefer to be assigned to a different station that would not involve viewing that area.

### Equity

The practice of financially compensating medical student ESPs may be construed as potentially coercive because medical students are a financially vulnerable population^24^ with few opportunities to earn money because of their rigorous coursework and schedules. Offering an experience with combined educational, professional, and small monetary compensation, however, may ultimately be a means of promoting multifactorial student wellness and benefit the students.

Case 1 also raises an equity in recruiting issue: Some ultrasound programs at our institution have historically had a practice of recruiting only “male models” for their educational events to avoid performing ultrasounds on ESPs whose chests and upper bodies would be more sensitive to expose. Defining who fits the criteria of “male” is problematic and is additionally inequitable as then only “male models” are offered the potential educational, professional, and financial gain from being an ESP. Further, it may be a disservice to the ultrasound learners and their future patients to restrict the educational experience to a certain subset of the population when they will need to be able to use their skills on any patient in the future. As in the discussion of sensitive exams, supporting ESP autonomy in these scenarios involves being transparent about the type of exams that will be done, outlining steps that will be taken to promote ESP dignity and comfort, and allowing ESPs to decide whether they are willing to participate.

## OBJECTIVES

In response to concerns raised at our institution illustrated by these 4 cases, we designed and implemented a system of protections for ESPs that incorporate the key ethical principles and frameworks described above.

## DESIGN

### Consent Form

In collaboration with legal counsel, the medical school’s standardized patient program, and a clinical ethicist, we created a best practice consent form for all activities involving human models for ultrasound learning. This document (Appendix 1) elaborates the relevant risks and benefits of ultrasound, including the risk of encountering incidental findings. Furthermore, the form describes the intent of the scanning exercise, the learning environment, the learner population, and potential gamification of the event. The ESPs are asked to specify whether they are pregnant or likely to be pregnant during the event given the theoretical risks that certain ultrasound applications may pose to fetuses.^25^ Among a list of options, ESPs are asked to mark which areas of the body they feel comfortable having examined. Contact information for one of the course leaders is also provided for them to direct any questions or concerns.

### Ombudsperson Role

To support the physical and psychological safety of ESPs, an ombudsperson role was implemented. The purpose of the ombudsperson is to serve as a neutral third party to whom ESPs can voice concerns in real time and who can advocate on the ESPs’ behalf. Specifically, the ombudsperson is designed to be a faculty or staff member who is neither part of ultrasound leadership nor involved in the coordination of the educational event to avoid conflict of interest and optimize approachability.

The ombudsperson is also assigned specific responsibilities and provided a tip sheet prior to the educational event. They are responsible for reviewing each ESP’s consent form before the event and ensuring the ESP’s assigned station aligns with the preferences indicated on their form. Embedded simulation participants are asked to arrive early to confirm their preferences verbally with the ombudsperson. The ombudsperson helps orient the ESP to the event by introducing them to their assigned instructor and their assigned station. They are present throughout the event, circulating among the ESPs to check in regularly. They need a clear reporting structure for addressing concerns as they arise. For example, the ombudsperson should be empowered to pause the learners as needed to intervene on learner behavior (ie, provide feedback on draping technique or amount of discomfort experienced by the ESP), facilitate change in station or assignment, or facilitate voluntary ESP withdrawal from the educational event.

Implementation of the ombudsperson role, while beneficial in many respects, can also carry challenges. Volunteers for the role should be trained in advance with the opportunity to ask clarifying questions. It may be difficult to recruit faculty volunteers who are not invested in ultrasound education. Furthermore, compensating a faculty ombudsperson may lead to prohibitive expense. Volunteers who have an adjacent relationship to the student population, such as administrative staff and faculty advisors, may be an excellent fit. Not only are they already invested in student education, but they also commonly have strong relationships with the students and are motivated to care for their concerns. Further, it is important to have ESP consent forms readily available to the ombudsperson on the day of the event to review with students physically. This redundancy helps prevent misunderstandings about consent and which studies will be appropriate.

### Regulation of Photography and Social Media Posting

Although we recognize the importance of social media in the era of virtual recruitment,^26^,^27^ given the risk of students having their photos taken in a potentially vulnerable circumstance (with bodily exposure for ultrasound examination), we changed the practice at our institution to more highly regulate photography during our educational events. Participants and instructors are specifically reminded to not include ESPs in any photos. We explicitly restricted photography during scanning activities and recommended that photography occur only at stations that do not use ESPs or in group photos before or after the event. We also encouraged our residency social media team to actively review photos to ensure no ESPs are present (even in the background) prior to interacting with event photos even if posted by others.

### Standardized Process for Identifying Incidental Findings

After being introduced to their assigned instructor, ESPs are prescanned for potential incidental findings before the educational event begins. This allows for private discussion and counseling of the ESP should any findings be encountered. It also allows for the ESP to be assigned a different station if they would prefer that discovery of or discussion of the incidental finding be completely avoided. The general incidental finding protocol used at our institution is available as Supplemental Appendix 1.

### Standardized Recruitment and Compensation Process

At our institution, we contact all medical students through a general call for volunteers via email listserv. An administrative assistant uses scripted language to describe the event and the roles and responsibilities of an ESP. Students are notified they will be compensated for their time with the compensation rate listed. Students contact the administrative staff member who then responds with a welcome email that details the types of ultrasounds that will be performed at the educational event, including the body parts that would be exposed and included in the exam. Our consent form is included. Students are given the opportunity to ask questions and to consent to any or none of the study types that will be performed. Once consent is received electronically, the students are added to the schedule and provided with appropriate logistical information.

## IMPACT AND EFFECTIVENESS

The revised consent form, ombudsperson role, and other interventions were implemented in January 2024 in the emergency department at a large academic medical center affiliated with a medical school. At the time of writing this manuscript, approximately 96 ESP volunteers have participated across 16 educational events coordinated by the department since implementation of these interventions. In addition to medical students, some volunteers were nurses, administrative staff, and physician assistants. All 96 volunteers signed the revised consent form.

Anecdotally, the revised consent form and presence of an ombudsperson have been positively received, with some participants noting that the consent form indicates a feeling of caring and that the department is serious about ESP comfort during educational events. Additionally, no photography concerns have arisen since implementation of these procedures.

One unexpected consequence of our interventions challenged us to be more thoughtful regarding ESP privacy. As part of the consent process, ESPs were asked to identify on a form what areas of the body they felt comfortable having examined. In the early stages of implementation, this form was visibly posted at the station the ESP was assigned to. This ensured the ombudsperson, trainees, and preceptors would not forget what the ESP had consented to. However, posting this information in a highly visible way inadvertently led to compromised ESP privacy and discomfort if the ESP felt they had to justify why they did not consent to certain exams. In response, this information was kept in a more discreet location where it was still readily available for reference.

## LIMITATIONS

This paper is limited to the experiences of a single institution, and we do not report a formal assessment of the effectiveness or perception of our developed interventions. Additional work could assess specific outcomes, including rates of ombudsperson intervention, station reassignment, ESPs declining examination of specific body regions, withdrawal from educational events, identification of incidental findings, and the impact of interventions on psychological safety using validated metrics. Further research can also explore medical student and ombudsperson perception of these implemented changes through survey or interview, the demographics of ESPs used by point-of-care ultrasound training programs across medical education, how these interventions and principles may evolve if ESP volunteers are not medical students (eg, if they are residents, fellows, faculty, or from other job families), and assess the implementation of these practices in different learning environments. An additional future direction for this work is the adaptation of these interventions to environments with fewer resources such as staffing and funding.

## CONCLUSION

There are many benefits to using medical student volunteers as embedded simulation participants; however, proactive measures are necessary to support the autonomy and safety of these volunteers. Motivated by real concerns raised by medical students at our institution, we created a comprehensive volunteer consent form, designed a novel ombudsperson role intended to support the safety and comfort of volunteers during educational events, and created formal restrictions surrounding photography and social media posts related to these events. While these perceived improvements are pending formal evaluation, our hope is that other institutions who use medical students as simulated patients will consider implementing similar practices to promote the psychological and bodily safety of these volunteers.

## Supplementary Information


