# Supplementary material for: Ethics of Medical Students as Embedded Simulation Participants in Ultrasound Education
Source: West J Emerg Med. 2026 Oct 18;27(6):1714–9. doi: 10.5811/westjem.68197 (PMC13762026; doi:10.5811/westjem.68197)
Supplement: Supplementary file 1 [file wjem-27-1714-s001.pdf]

Supplemental Appendix 1. Protocol for Addressing Incidental Findings in Educational Settings

PURPOSE: An incidental ultrasound finding is defined as a finding of any description that is perceived by the emergency medicine physician to be of concern to the patient while using the ultrasound machine for educational purposes or an unrelated clinical application. This policy creates a guideline for the management of said findings in the clinical environment.

STANDARDS:

1. All scans performed and saved will be reviewed weekly by the clinical ultrasound faculty to follow up on incidental findings not recognized on the initial ultrasound examination.
2. In the setting where a provider discovers a finding outside the scope of the emergency medicine credentialing package, the decision-making tree described under procedure (below) will be followed.

PROCEDURE

Upon observation of an incidental findings, the provider performing the ultrasound should:

- Seek input from providers with more clinical ultrasound experience (if available) to further delineate and/or review the incidental finding.
- Follow the standard policy of saving educational files with both patient name, MRN, and the provider's name.
- Email *** for more rapid QA of the scan in question.
- Identify which category of concern the incidental finding best fits and follow the recommended actions outlined below.

Concern Level 1 – Immediate Concern. If the finding is felt to present a threat to life and/or limb (including pregnancy), action is required immediately. Findings should be communicated to the clinical team caring for the patient. The clinical team should then obtain follow up imaging relevant to the findings.

- Example 1: The incidental finding of a suspected pregnancy should prompt further workup as medically indicated.
- Example 2: A mass is visualized in the left ventricle on an educational echo; the clinical team should be informed, and a cardiology-based echo should be obtained.

Concern Level 2 – Urgent Concern. These findings represent an acute or chronic pathology outside the scope of the provider's practice that is non-life threatening but is still anticipated to require close follow up. All Level 2 Incidental Findings should be emailed to *** for more rapid QA of the scan in question. Please defer to the clinical ultrasound faculty for further follow up on the issue.

- Example 1: During a FAST exam due to an MVC, the R kidney is noted to have a simple renal cyst.
- Example 2: While evaluating the IVC for overall fluid status, the gallbladder is noted to have stones; the patient is asymptomatic.
